# Supplementary material for: Content-rich biological network constructed by mining PubMed abstracts
Source: BMC Bioinformatics. 2004 Oct 8;5:147. doi: 10.1186/1471-2105-5-147 (PMC528731; doi:10.1186/1471-2105-5-147)
Supplement: Additional File 5 — The original Chilibot query results of the term "long-term potentiation (LTP)" and 22 other terms, limiting the latest references analyzed to the years 1990, 1995, 2000, and 2004. [file 1471-2105-5-147-S5.bz2 › chilibotAdditionalFile5/ltp2004/html/ERK_SYNAPTOPHYSIN.html]

 


 **ERK** and **SYNAPTOPHYSIN** 
  
Found 4 abstracts in PubMed,  **4 abstracts were retrieved and analyzed**.  


---

 Search Google  |
 PDF files only 
|  EDU domain only 

---

**Interactive relationship** (e.g. stimulation, inhibition, etc)

**Parallel relationship** (e.g. studied together, co-existance, homology, etc.)

- The extracellular regulated protein kinases  **ERK**  and  **synaptophysin** , proteins implicated in exocytosis, are both tyrosine phosphorylated by bpV phen in a dose and time dependent manner, with a maximal effect at 30 microM.  Ref: 11792460 Neurochem Int, 2002
- These results suggest that this peroxovanadium complex enhances dopamine exocytosis, at least in part, by  **ERK**  mediated signaling pathway and  **synaptophysin**  associated phosphatase s.  Ref: 11792460 Neurochem Int, 2002
